# Can adolescents' subjective wellbeing facilitate their pro-environmental consumption behaviors? Empirical study based on 15-year-old students

**DOI:** 10.3389/fpubh.2023.1184605

**Published:** 2023-10-05

**Authors:** Min Zhang, Weidong Zhang, Yong Shi

**Affiliations:** ^1^The Co-innovation Center for Social Governance of Urban and Rural Communities in Hubei Province, Zhongnan University of Economics and Law, Wuhan, Hubei, China; ^2^School of Business Administration, Zhongnan University of Economics and Law, Wuhan, Hubei, China; ^3^School of Accounting, Zhongnan University of Economics and Law, Wuhan, Hubei, China

**Keywords:** subjective wellbeing, pro-environmental consumption behaviors, adolescent, life satisfaction, positive emotions, negative emotions

## Abstract

**Purpose:**

To address the challenge of declining pro-environmental behaviors in adolescence, this paper uses the theoretical foundations for subjective wellbeing to verify the influence of three latent dimensions of subjective wellbeing (life satisfaction, positive emotions, and negative emotions) on adolescents' pro-environmental consumption behaviors (PECBs). Furthermore, we explore the moderating effects of nations and regions in the relationship between subjective wellbeing and PECBs in adolescents.

**Method:**

Based on the international data from the Programme for International Student Assessment 2018 (PISA 2018), we construct a unique dataset that includes 57,182 samples related to the subjective wellbeing and PECBs of 15-year-old students from eight countries/economies. Specially, we employ an ordered probit model to test our hypotheses.

**Findings:**

Both adolescents' life satisfaction and positive emotions can significantly improve their PECBs, while there is a significant negative association between negative emotions and PECBs. At the nation's level, adolescents' life satisfaction and negative emotions in developed countries/economies significantly impact PECBs. In contrast, the positive emotions of adolescents in developing countries/economies have more substantial effects on PECBs. At the regional level, the impact of all three dimensions of adolescents' subjective wellbeing on PECBs is more significant in rural than urban areas.

**Originality/value:**

This paper provides a new perspective for understanding adolescents' PECBs from the insights of subjective wellbeing. Previous studies have examined the effects of life satisfaction or happiness on PECBs in adults. This paper examines the impact of subjective wellbeing on adolescents' PECBs from life satisfaction, positive emotions, and negative emotions, which suggests that promoting adolescents' subjective wellbeing can be an effective strategy for encouraging PECBs. From a comparative research perspective, we further analyze the differences between the nations at different levels of development, the rural and urban areas, providing a valuable reference for policymakers and practitioners in promoting pro-environmental behaviors among adolescents.

## 1. Introduction

Adolescents and youth are the largest generations in history, at over 1.8 billion (1). As the un claimed, “it is impossible to achieve the Sustainable Development Goals (SDGs) by 2030 without the active participation of the largest generation of changemakers” (2). The choices adolescents make today—or the lack of them—will have a profound impact on the planet and on our survival as a species (3). However, numerous studies have shown that adolescents' interest in environmental protection presents a decreasing trend in adolescence relative to childhood and adulthood (4–12). How to promote adolescents' pro-environmental behaviors, then, is of increasing concern to policy-makers and educators (13–16).

Pro-environmental consumption behaviors (PECBs) are actions taken by individuals that prioritize environmental sustainability in their consumer choices (17, 18). A large body of prior literature on the influence of adolescent PECBs has emphasized the impact of external factors, such as family and peer relationships (19–24), social norms (25, 26), nature or socio-spatial distance (5, 27–30), and environmental knowledge (31–33). Other scholars have investigated the influence of internal factors, for instance, adolescents' personality traits (34, 35) and environmental concerns (32, 36–39). It is thus clear that previous literature has focused on objective predictors that influence adolescents' PECBs, while the potential influence of adolescents' subjective feelings on PECBs remains to be explored (18, 40).

Research in positive psychology suggests that those with higher levels of subjective wellbeing are more willing to give more money or time to engage in socially beneficial behavior, while those with lower levels of subjective wellbeing are often under urgent pressure to make the modest lives and are overwhelmed by the need to defend the public interests (41–43). But is this the case in the field of PECBs?

Some scholars suggest that happier people are more willing to pay more or change their consumption behaviors to protect the environment (17, 18, 40, 42, 44–48). However, the result has been only empirically analyzed in adults. Adolescence is a critical stage of human development between childhood and adulthood (49). Numerous studies have revealed a declining trend in interest in environmental issues among adolescent groups (8, 9, 11, 12, 25, 50), a phenomenon known as“time out”(12) or “adolescent dip” (11). Can, then, adolescents' subjective wellbeing promote their PECBs as well as adults?

In response to a growing call for research, several scholars have explored whether adolescents' increased happiness or life satisfaction would fare better in PECBs (51). Nonetheless, two gaps in the existing literature still need to be addressed.

On the one hand, although a growing body of literature has focused on the relationship between subjective wellbeing and PECBs, subjective wellbeing is regarded as a one-dimensional structure in the existing literature (18). Subjective wellbeing, wellbeing, happiness, life satisfaction, and positive emotions are often used interchangeably (40, 44, 45). In reality, there is a large consensus that life satisfaction and emotions are two fundamental building blocks of subjective wellbeing (41, 52, 53). In this vein, subjective wellbeing includes both an affective component (positive and negative emotions) and a cognitive component (one's judgment of one's overall life satisfaction or satisfaction with specific domains of one's life) (14, 43, 52). That is, subjective wellbeing contains three related but independent components: life satisfaction, positive emotions, and negative emotions (43, 54–56). Previous research has focused on the effects of life satisfaction or positive emotions on PECBs, neglecting the impact of negative emotions. Moreover, extant research suggests negative emotions or unhappiness can also predict pro-social behaviors (43). Hence, this paper will examine whether subjective wellbeing is associated with adolescents' PECBs along three dimensions: life satisfaction, positive emotions, and negative emotions to address the first research gap.

On the other hand, the existing research that has been conducted usually focuses on one country or region, and there is a lack of international and cross-regional comparative studies. Moreover, extensive research has focused on developed countries, whereas research on those in developing countries has yet to be explored, especially the comparative studies during different development levels of nations. In addition, comparative studies of PECBs in adolescents in different areas, such as urban and rural areas, remain a puzzle.

To shed light on these puzzles, using a sample of 57,182 adolescents across eight countries/economies, we explore whether adolescents' PECBs are associated with all of the three components of subjective wellbeing by the ordered probit model. Furthermore, we explore the differences between developing and developed countries/economies, and between rural and urban areas.

This paper is structured as follows. Section 2 presents the literature overview of subjective wellbeing and PECBs and proposes the hypotheses. In Sections 3, we describe the data sources, variables, and empirical model. Section 4 reports the general results and the mechanism analysis of the moderating effects. In Section 5, we further discuss the findings. Finally, conclusions are drawn in Section 6.

## 2. Literature review

### 2.1. Adolescents' PECBs

Adolescents' lifetimes, in particular, will face the environmental consequences of actions taken or not taken today. Their active engagement in understanding the state of our environment (and the importance of protecting it) is critical to achieving sustainable development globally (3). PECBs are the human behaviors that mitigate various environmental issues, including climate change, environmental pollution, and the loss of biodiversity (57–59). Precisely, PECBs comprise the committing of acts that benefit the ecological environment (e.g., recycling, use of non-toxic substances, changing purchasing behaviors) and the omission of actions that harm it (e.g., avoiding air travel, reducing waste production, minimizing energy consumption) (17, 60, 61).

For adolescents, United Nations Environment Programme (UNEP) suggests they can influence the market by buying or not buying (3). PECBs and public environmental protection activities are the main ways adolescents protect the environment. PECBs are more related to the private lifestyle of adolescents. Nevertheless, adolescents may not have sufficient exposure or knowledge of issues related to environmental degradation, and therefore may not see the relevance of these issues to their daily lives (27, 62). More specifically, as students, adolescents are surrounded by personal and academic pressures such as grades, social life, and extracurricular activities, which makes it difficult for them to devote more money or time to environmental issues. The decline in interest in environmental issues among adolescents is a tremendous challenge for the earth's future that may require a more effective approach to address (3, 63).

### 2.2. Life satisfaction and PECBs in adolescents

Life satisfaction is a subjective measure of one's overall sense of fulfillment and contentment with their life (41, 45, 54). It is people's evaluation of their life as a whole, including their past, present, and future (64, 65). Previous studies have shown that life satisfaction significantly impacts PECBs (43, 45). Wang and Kang (45) argued that people with higher life satisfaction performed better in PECBs. Ones with high life satisfaction often have a strong sense of pro-society values, including a concern for social and environmental issues (43). This value system can lead them to choose environmentally friendly products and services, even if they cost more or are less convenient (40, 45).

Moreover, individuals with high life satisfaction may be more likely to be influenced by social norms that promote PECBs. Ouyang et al. (44) suggested that people might feel responsible for their communities and want to be seen as environmentally responsible and socially conscious. In this vein, ones may support companies that prioritize sustainable practices and ethical sourcing even if they may pay more (40). In contrast, those less satisfied with their lives are likely to be preoccupied with improving their situation, which can overshadow concerns about social issues (18). Therefore, we propose the following hypothesis:

*H1a. Adolescents' life satisfaction is significantly and positively correlated with their PECBs*.

### 2.3. Positive emotions and PECBs in adolescents

Positive emotions are subjective experiences that are typically pleasant and enjoyable. These emotions include feelings such as happiness, joy, contentment, gratitude, and hope (55, 56). Research has shown that cultivating positive emotions can benefit individuals and society; for example, positive emotions can motivate people to engage in pro-environmental behaviors that protect the natural environment and preserve its resources (40, 44, 66, 67).

Notably, the influence of positive emotions on PECBs has been identified in the literature. Yuan (68), Briones et al. (69) suggested that positive emotions were associated with greater self-control and regulation, which could motivate people to act in ways that align with their values and beliefs. This could also translate into PECBs, such as recycling or reducing energy consumption. In the opinions of Meinhold and Malkus (32), Kaida and Kaida (70), people who experience positive emotions may be more likely to conform to social norms related to sustainability, especially if those norms are reinforced by positive feedback from others. Therefore, they may be more likely to consider the environmental impact of their actions and choose PECBs. Besides, people with more positive emotions usually have a stronger sense of altruism and reciprocal cooperation (44). This can lead to increased concern for the environment and a desire to engage in behaviors that benefit others, even if it requires personal sacrifice or inconvenience (40). Therefore, we propose the following hypothesis:

*H1b. Adolescents' positive emotions are significantly and positively correlated with their PECBs*.

### 2.4. Negative emotions and PECBs in adolescents

Negative emotions are subjective feelings that can cause one to be miserable and sad, such as hate, afraid, anger, jealousy, and sadness (56, 67). Negative emotions may dampen people's enthusiasm for life and reduce their confidence and self-esteem (71). In this context, negative emotions may stop one from thinking and behaving rationally and seeing situations from their proper perspective. Adolescence is a period of dramatic development. The frontal lobes have not entirely developed, which causes immature self-emotional management skills (72, 73). Adolescents in this period are more concerned with their inner feelings. When immersed in negative emotions, it is a big challenge for them to pay attention to social and public issues and take social responsibility. Erikson (74) referred to these irresponsible behaviors of adolescents as “adolescence moratoriums.”

Although research has suggested that negative emotions, through fear, anger, guilt, shame, etc., help to evoke environmentally responsible behaviors (67). In fact, using negative emotions to drive behavior change limits our attention to the short term. As Carter (67), Sheldon and Kasser (75) argued that negative emotions might lead to a focus on immediate needs and desires rather than long-term consequences. For example, someone feeling stressed or anxious may prioritize convenience over sustainability, choosing disposable products instead of reusable ones to save time and effort (76). Moreover, the negative emotions that come with psychological threats can motivate one to give more attention to external goals, which may cause lower PECBs (75, 76). Specifically, when people are threatened regarding survival, finances, or relationships, they are more inclined toward extrinsic goals such as financial success, popularity, and image than intrinsic goals such as personal growth, social norms, and community contributions (75–78). Thus, we propose the following hypothesis:

*H1c. Adolescents' negative emotions are significantly and negatively correlated with their PECBs*.

### 2.5. Moderating effects: developing or developed countries/economies

Nations worldwide are usually divided into developed and developing countries/economies based on their economic development, wealth, and wellbeing. The level of development of a country or economy is linked to the long and healthy life, access to knowledge, and standard of living of its people, which are also significant predictors of PECBs (40, 79).

In developed countries/economies with high levels of material wealth and availability of resources, people might be more selective about the products they purchase (46, 47, 80). More specifically, individuals in developed countries/economies tend to have a higher living standard, enabling them to make income sacrifices to change their purchase behaviors for environmental sustainability (40, 42). Additionally, adolescents in developed countries have greater access to education, information, and media resources, which can increase their awareness and concern about environmental issues.

Conversely, in developing countries/economies where poverty, inequality, and environmental degradation are pressing issues, individuals' subjective wellbeing may be more tied to stable access to basic needs of survival (46, 80). They may not be more selective in their consumption behaviors, and their financial constraints may limit their choices (41). Furthermore, adolescents in developing countries might lack exposure to such information, so their awareness and interest in environmental problems may be lower. Therefore, the impact of adolescents' subjective wellbeing on their PECBs should differ between developing and developed countries/economies due to different economic and social development levels. Based on the above analysis, we propose the following hypotheses:

*H2a. Compared to the group of developing countries/economies, the effect of adolescents' life satisfaction on PECBs is more substantial in developed countries/economies*.

*H2b. Compared to the group of developing countries/economies, the effect of adolescents' positive emotions on PECBs is more substantial in developed countries/economies*.

*H2c. Compared to the group of developing countries/economies, the effect of adolescents' negative emotions on PECBs is more substantial in developed countries/economies*.

### 2.6. Moderating effects: the rural or urban areas

Just as there are significant differences in levels of development between countries, there are essential differences between rural and urban areas in terms of population density, economic activity, infrastructure, lifestyle, education, health care, and the environment (81, 82).

In rural areas, communities have stronger social connections and a sense of community compared to urban areas. Ouyang et al. (44) argued that residents in rural areas lived in a society of acquaintances, and participation in environmental protection helped to strengthen their social capital and network of relationships. Besides, rural adolescents may have a closer relationship with their local environment and natural surroundings than urban adolescents (30). Specifically, rural adolescents may be more involved in farming, gardening, or outdoor activities. In contrast, the barriers to opportunities for adolescents in urban areas to connect with nature while enjoying high industrialization and modernization levels are increasing. Solano-Pinto et al. (51) have empirically tested whether there is a reciprocal influence between children's wellbeing and connectivity with nature which may promote their PEBs. Thus, the effect of adolescents' subjective wellbeing on PECBs should be more pronounced among adolescents in rural areas than in urban areas. Based on the above analysis, we propose the following hypotheses:

*H3a. Compared to the group in the urban area, the effect of adolescents' life satisfaction on PECBs is more substantial in rural regions*.

*H3b. Compared to the group in the urban area, the effect of adolescents' positive emotions on PECBs is more substantial in rural regions*.

*H3c. Compared to the group in the urban area, the effect of adolescents' negative emotions on PECBs is more substantial in rural regions*.

## 3. Methodology

### 3.1. Data

The data for this paper comes from two different sources. One is the individual dimension data, 2018 Programme for International Student Assessment (PISA 2018), from Organization for Economic Co-operation and Development (OECD). The other is the national dimension data, the human development index (HDI), from the United Nations Development Programme (UNDP).

The questionnaire from PISA 2018 is a comprehensive and rigorous international standardized assessment for adolescents, with both the multi-item measurements of subjective wellbeing and PECBs (14). PISA 2018, the triennial assessment launched in 1997 by OECD, is the latest wave of surveys. PISA 2018 assesses the extent to which 15-year-old students have acquired knowledge and skills to meet real-life challenges near the end of their compulsory education, conducted in eighty countries/economies worldwide. Unfortunately, the relevant questions for subjective wellbeing and PECBs are surveyed in only eight countries/economies in PISA 2018. Excluding invalid responses, 57,182 samples constitute the dataset for this study.

### 3.2. Variables

#### 3.2.1. Dependent variable: PECBs

In the PISA 2018 student questionnaire, three question items are related to PECBs (see **Table 3**). Respondents are asked whether they have participated in PECBs in the questionnaire. We assign a value to each question answer. “No” is given a matter of “0”; “yes” is given a value of “1”.

#### 3.2.2. Independent variable: subjective wellbeing

Subjective wellbeing is the core set of explanatory variables with three dimensions (see **Table 3**). Life satisfaction is measured by the four-point Likert scale of 10 question items, ranging from 1 (Not at all satisfied) to 4 (Totally satisfied). Positive emotions are measured by the four-point Likert scale of five question items, ranging from 1 (Never) to 4 (Always). Negative emotions are measured by the four-point Likert scale of four question items, ranging from 1 (Never) to 4 (Always). All three scales are tested reliability and validity. Cronbach's α for these scales are all >0.8, indicating that the reliability of scales is acceptable (see Table 1). The KMO value and Bartlett's test of sphericity show satisfactory results (Life satisfaction: KMO = 0.917; χ^2^ = 246,570.980, *p* < 0.001; positive emotions: KMO = 0.853; χ^2^ = 113,166.304, *p* < 0.001; negative emotions: KMO = 0.750; χ^2^ = 156,573.700, *p* < 0.001), which indicate the suitability of factor analysis. Factor loadings (FL) of each item above 0.5 indicate good construct validity (see Table 1). Both the average variance extracted (AVE) of positive emotions and negative emotions are above 0.50, while the AVE of life satisfaction is 0.482 (see Table 1). However, the composition reliability (CR) values of the three constructs are all above 0.60 (see Table 1), demonstrating the convergent validity is acceptable (83).

**Table 1:** Reliability and validity of the measurement scale of subjective wellbeing.

**Variable**	**Measure items**	**FL**	**Cronbach's α**	**CR**	**AVE**
Life satisfaction (X1)	Your health	0.647	0.891	0.903	0.482
	The way that you look	0.625			
	What you learn at school	0.684			
	The friends you have	0.690			
	The neighborhood you live in	0.705			
	All the things you have	0.747			
	How you use your time	0.674			
	Your relationship with your parents/guardians	0.699			
	Your relationship with your teachers	0.718			
	Your life at school	0.722			
Positive emotions (X2)	Happy	0.805	0.835	0.832	0.560
	Lively	0.750			
	Proud	0.533			
	Joyful	0.833			
	Cheerful	0.826			
Negative emotions (X3)	Scared	0.780	0.866	0.764	0.570
	Miserable	0.697			
	Afraid	0.792			
	Sad	0.727			

Additionally, we test the discriminant validity. Table 2 shows that the square root of the AVE for each construct is all greater than its correlation with the other variables, demonstrating the discriminant validity of the measures (18).

**Table 2:** The discriminant validity of the measures.

**Variables**	**Life satisfaction**	**Positive emotions**	**Negative emotions**
Life satisfaction	*0.694*		
Positive emotions	0.473	*0.748*	
Negative emotions	−0.335	−0.287	*0.755*

Numbers in the diagonal and italics are the square roots of the AVEs, while others are correlations between variables.

#### 3.2.3. Moderating variables: the nation and region

In this paper, the samples are drawn from eight countries/economies: Bulgaria, Hong Kong, Ireland, Mexico, Panama, Serbia, Spain, and United Arab Emirates (the UAE). In order to test the moderating effect of the level of development of countries or economies, we classify them as developing countries/economies and developed countries/economies. The HDI, produced by UNDP since 1990, consists of three components: a long and healthy life, access to education, and a decent standard of living (84). The HDI covers both economic and social indicators, replacing the single GDP per capita measurement system and providing a more comprehensive and scientific picture of the nation's level of development. The HDI ranges from 0 to 1, with higher values being better. The four subgroups are as follows: less than 0.550 for low development; between 0.550 and 0.699 for medium development; 0.700 and 0.799 for high development; and ≥0.800 for extremely high development.

To explore the impact of the level of national development on the PECBs of 15-year-olds, we set the data collection period from birth to the year the adolescents were surveyed, i.e., 2004 to 2018. We take the average value of the HDI for each country or economy over a total of 15 years, from 2004 to 2018. The results show that Hong Kong, Ireland, Spain, and the UAE have a mean HDI above 0.800 for these 15 years, while Bulgaria, Mexico, Panama, and Serbia have HDIs between 0.700 and 0.799. According to existing studies (84), HDI above 0.800 is considered the developed countries or economies, while the rest are developing countries or economies. Therefore, in this paper, the group with a high HDI is regarded as a developing country/economy and set a value of 0. The group with a very high HDI is considered a developed country/economy and assigned a value of 1.

As for the regions, in PISA 2018, the communities in which students' schools are located are divided into five categories by population (see Table 3). We further divide them into the rural (Assign a value of 0) and urban areas (Assign a value of 1).

**Table 3:** Descriptive statistics.

**Variable**	**Definition**	**N**	**SD**	**Mean**	**Min**	**Max**
PECBs No=0;Yes=1.	Reducing energy use	57,182	0.435	0.747	0	1
	Paying more for environmental products	57,182	0.500	0.483	0	1
	Boycotting products or companies for environmental reasons	57,182	0.456	0.295	0	1
Life satisfaction Not at all satisfied=1;Not satisfied=2;Satisfied=3;Totally satisfied=4.	How satisfied are you with each of the following?					
	Your health	57,182	0.738	3.162	1	4
	The way that you look	57,182	0.802	2.966	1	4
	What you learn at school	57,182	0.761	2.922	1	4
	The friends you have	57,182	0.692	3.326	1	4
	The neighborhood you live in	57,182	0.757	3.159	1	4
	All the things you have	57,182	0.670	3.337	1	4
	How you use your time	57,182	0.810	2.930	1	4
	Your relationship with your parents/guardians	57,182	0.765	3.265	1	4
	Your relationship with your teachers	57,182	0.751	3.015	1	4
	Your life at school	57,182	0.769	3.030	1	4
Positive emotions Never=1;Rarely=2; Sometimes=3; Always=4.	How often do you feel as described below?					
	Happy	57,182	0.644	3.434	1	4
	Lively	57,182	0.716	3.265	1	4
	Proud	57,182	0.800	2.999	1	4
	Joyful	57,182	0.702	3.352	1	4
	Cheerful	57,182	0.688	3.380	1	4
Negative emotions Never=1;Rarely=2; Sometimes=3; Always=4.	How often do you feel as described below?					
	Scared	57,182	0.786	2.215	1	4
	Miserable	57,182	0.848	2.239	1	4
	Afraid	57,182	0.849	2.494	1	4
	Sad	57,182	0.752	2.529	1	4
Gender	Male=0; Female=1	57,182	0.500	0.516	0	1
Grade	Student International Grade	57,182	0.667	9.756	7	12
Environmental knowledge	Explaining how carbon-dioxide emissions affect global climate change (I couldn't do this=1; I would struggle to do this on my own=2; I could do this with a bit of effort=3; I could do this easily=4)	57,182	0.946	2.800	1	4
Region	Which of the following definitions best describes the community in which your school is located? (Rural area=0; urban area=1)	57,182	0.239	0.939	0	1
NationHuman Development Index(HDI)	The mean value of HDI in the respondent's country or economy from 2004 to 2018. (high = 0; extremely high = 1)	57,182	0.413	0.781	0	1

#### 3.2.4. Control variables

The individual demographic characteristics, such as gender and grade, are controlled. Moreover, according to Ouyang et al. (44), we further hold for the effect of their environmental knowledge. Table 3 shows the above variables' descriptive statistics in detail.

### 3.3. Empirical model

Given that the dependent variable PECBs is an ordered variable, according to Ouyang et al. (44), Wang and Kang (45), we use an ordered probit model to test the effect of subjective wellbeing on PECBs. The model is as follows:


(1) $$\begin{array}{l} PECBs_{i}=\beta_{0}+\beta_{1}Subjectivewellbeing_{i}+\beta_{2}X_{i}+\varepsilon_{i\;} \end{array}$$

where *i* represents the individual. *PECBs*_*i*_, the dependent variable, stands for the PECBs of individual *i*. *Subjectivewellbeing*_*i*_, the set of the core explanatory variables, represents the subjective wellbeing of individual *i*. *X*_*i*_ is the set of control variables. Lastly, ε_*i*_ is an error term.

## 4. Results

### 4.1. General results

The regression results of the effect of the three dimensions of subjective wellbeing on PECBs in adolescents are reported in Table 4. The results show that adolescents' life satisfaction and positive emotions are significantly positively associated with their PECBs, while negative emotions are significantly negatively associated with their PECBs. Thus, H1a, H1b, and H1c are all verified.

**Table 4:** Benchmark regression.

	**PECBs (1)**	**PECBs (2)**	**PECBs (3)**
Life satisfaction	0.142^***^ (15.43)		
Positive emotions		0.080^***^ (9.18)	
Negative emotions			−0.069^***^ (−8.74)
Grade	0.099^***^ (14.05)	0.106^***^ (15.03)	0.107^***^ (15.16)
Gender	−0.046^***^ (−5.09)	−0.051^***^ (−5.63)	−0.033^***^ (−3.63)
Environmental knowledge	0.123^***^ (24.85)	0.127^***^ (25.59)	0.131^***^ (26.45)
Observations	57,182	57,182	57,182
Pseudo R-squared	0.009	0.008	0.008

*** p < 0.001 and z-values in parentheses.

Furthermore, at the individual level, the grade level, gender, and environmental knowledge of adolescents all significantly impact PECBs. The research sample for this paper is 15-year-old school students of the same age and in grades ranging from 7th to 12th. The results show that the higher the grade level of the adolescents, the better their performance on PECBs. At the same time, there is a significant positive correlation between adolescents' environmental knowledge and PECBs. The findings indicate that the promotion of environmental knowledge and academic knowledge helps adolescents to develop sustainable consumption behaviors that are conducive to environmental protection, which is consistent with Carmi et al. (85), Latif et al. (86), Liu et al. (87), Meyer (88). Moreover, some studies have shown that females show stronger environmental attitudes and behaviors than males (89–91). However, our results show that girls perform weaker than boys in PECBs, which is consistent with the findings of Vicente-Molina et al. (92).

### 4.2. Moderating effects

We further examine whether the impact of adolescents' subjective wellbeing on PECBs differs between developing and developed countries/economies. At the same time, we test the significance of the differences between the three groups. The results show that the differences between all three groups are significant at the 0.001% level (see *Empirical p-value* in Table 5). The results show that the positive impact of increased life satisfaction on adolescents' PECBs is more substantial in developed countries/economies compared (*β* = 0.173 , *p* < 0.001) to developing countries/economies (*β* = 0.047, *p* < 0.01). Thus, H2a is verified.

**Table 5:** Moderating effect of the nation.

	**(1)**	**(2)**	**(3)**	**(4)**	**(5)**	**(6)**
	**Developing**	**Developed**	**Developing**	**Developed**	**Developing**	**Developed**
Life satisfaction	0.047^*^	0.173^***^				
	(2.55)	(16.31)				
Positive emotions			0.120^***^	0.069^***^		
			(6.42)	(6.95)		
Negative emotions					0.007	−0.092^***^
					(0.46)	(−9.99)
Grade	0.054^**^	0.108^***^	0.050^**^	0.116^***^	0.056^**^	0.115^***^
	(3.14)	(13.49)	(2.90)	(14.65)	(3.26)	(14.43)
Gender	−0.117^***^	−0.025^*^	−0.117^***^	−0.032^**^	−0.121^***^	−0.009
	(−6.11)	(−2.50)	(−6.10)	(−3.13)	(−6.13)	(−0.88)
Environmental Knowledge	0.111^***^ (10.01)	0.127^***^ (22.79)	0.105^***^ (9.50)	0.132^***^ (23.78)	0.114^***^ (10.35)	0.136^***^ (24.39)
Observations	12,497	44,685	12,497	44,685	12,497	44,685
Pseudo R-squared	0.005	0.011	0.007	0.009	0.005	0.009
Empirical p-value	0.000^***^		0.011^*^		0.000^***^	

*** p < 0.001,

** p < 0.01,

* p < 0.05, and z-values in parentheses.

The effect of negative emotions on PECBs also confirms this result. The significant inhibitory effect of negative emotions on PECBs is more strongly manifested in developed countries/economies (*β* = −0.092, *p* < 0.001). Nevertheless, the impact of negative sentiment on PECBs is not significant in developing countries/economies. Thus, H2c is verified. Notably, on the dimension of positive emotions, adolescents' subjective wellbeing in developing countries/economies (*β* = 0.120, *p* < 0.001) has more substantial positive impacts on their PECBs than developed countries/economies (*β* = 0.069, *p* < 0.001). Thus, H2b is not verified.

On the other hand, we explore the moderating effect of region on adolescents' subjective wellbeing on their PECBs. Table 6 shows that the significant effects of subjective wellbeing on PECBs are all stronger for adolescents in rural areas than in urban areas. Moreover, we test the significance of the differences between the three groups. The results show that the differences between all three groups are significant at the 0.001% level (see *Empirical p-value* in Table 6). Thus, H3a, H3b, and H3c are all verified.

**Table 6:** Moderating effect of region.

	**(1)**	**(2)**	**(3)**	**(4)**	**(5)**	**(6)**
	**Rural**	**Urban**	**Rural**	**Urban**	**Rural**	**Urban**
*Life satisfaction*	0.282^***^	0.129^***^				
	(7.87)	(13.54)				
*Positive emotions*			0.189^***^	0.072^***^		
			(5.25)	(7.92)		
*Negative emotions*					−0.217^***^	−0.055^***^
					(−6.85)	(−6.77)
*Grade*	0.169^***^	0.094^***^	0.192^***^	0.100^***^	0.188^***^	0.101^***^
	(6.02)	(12.84)	(6.92)	(13.64)	(6.78)	(13.77)
*Gender*	−0.047	−0.048^***^	−0.038	−0.053^***^	0.006	−0.039^***^
	(−1.26)	(−5.19)	(−1.02)	(−5.75)	(0.15)	(−4.13)
*Environmental* *Knowledge*	0.135^***^ (6.73)	0.123^***^ (24.05)	0.143^***^ (7.17)	0.127^***^ (24.70)	0.153^***^ (7.68)	0.130^***^ (25.42)
*Observations*	3,480	53,702	3,480	53,702	3,480	53,702
*Pseudo R-squared*	0.022	0.009	0.018	0.008	0.020	0.008
*Empirical p-value*	0.000^***^		0.000^***^		0.000^***^	

*** p < 0.001 and z-values in parentheses.

## 5. Discussion

The results of this paper show that life satisfaction and positive emotions are significantly associated with greater PECBs. In contrast, negative emotions are associated considerably with lower PECBs in adolescents, even after controlling for environmental knowledge and sociodemographic variables. There are consistently significant correlations between the three dimensions of subjective wellbeing and PECBs in developed countries/economies. However, in developing countries/economies, life satisfaction and positive emotions significantly affect PECBs, while negative emotions do not correlate substantially with PECBs. Interestingly, the significant impact of adolescents' subjective wellbeing on PECBs is more substantial in developing countries/economies than in developed countries/economies. More specifically, in developed countries/economies, adolescents' life satisfaction and negative emotions significantly impacted their PECBs more than in developing countries/economies. However, the positive sentiment of adolescents in developing countries/economies has a more significant impact on PECBs than in developed countries/economies.

Furthermore, life satisfaction, positive emotions, and negative emotions are all significantly associated with PECBs in rural and urban areas. Notably, all these significant correlations are more substantial in rural than urban areas. The results indicate that adolescents in rural areas may have more opportunities to participate in community-based activities and get close to nature, which promotes sustainability and environmental protection. This can give them a sense of purpose and fulfillment, which may further reinforce their commitment to pro-environmental behaviors.

### 5.1. Theoretical implications

These findings contribute to the existing literature in several ways. Firstly, our study offers new ideas for approaching the challenge of declining environmental behavior in adolescents (11, 12). Our results confirm that subjective wellbeing, as an excellent predictor of PECBs, is not only in adults but also in adolescents, which builds on previous research examining the association between subjective wellbeing and pro-environmental behaviors in adults (18, 40, 44, 45). Secondly, our results also provide new evidence for developing the theory of subjective wellbeing. Building on the relationship between the three dimensions of subjective wellbeing (life satisfaction, positive emotions, and negative emotions) on pro-social behavior in adults by Kushlev et al. (43), our results further confirm that all three components of subjective wellbeing are significantly correlated with PECBs in adolescents. Last but not least, this paper broadens positive psychology from a comparative perspective. The significant differences between developed and developing nations, and between rural and urban areas, provide essential and concrete references for policymakers to enhance PECBs for adolescents.

### 5.2. Practical implications

The finding that subjective wellbeing positively influences PECBs has important practical implications for promoting sustainable consumption and environmental protection. This paper contributes to understanding the psychological mechanisms underlying environmentally friendly behaviors. It suggests that promoting subjective wellbeing can be an effective strategy for encouraging PECBs in adolescents. Our findings also highlight the importance of fostering subjective wellbeing as a critical component of sustainable consumption policies and programs. By improving adolescents' subjective wellbeing, policymakers and practitioners can create an environment that fosters pro-environmental attitudes and behaviors, ultimately leading to more sustainable lifestyles and a healthier planet.

More specifically, in developed countries/economies, more attention should be paid to the positive emotions of adolescents. In comparison, in developing countries/economies, more attention should be paid to the life satisfaction of adolescents and the impact of negative emotions on PECBs. Additionally, focusing on PECBs for adolescents in urban areas is essential. Policymakers, schools, and families should create more opportunities for young people to connect with nature and participate in environmental activities.

### 5.3. Limitations and further research

The limitations should also be considered when interpreting the results of this paper. Firstly, our study uses cross-sectional data and could not provide any evidence of causality. There are few international research projects on adolescents' subjective wellbeing and pro-environmental behavior. We look forward to more long-term follow-up projects to examine the causal relationship and long-term effects between adolescents' subjective wellbeing and PECBs. The second limitation concerns the measurement of adolescent PECBs used in this paper are self-reported rather than actual pro-environmental behaviors. Moreover, all PECBs are measured on dichotomous yes or no scales. Future research would benefit from a more detailed classification and more accurate frequency measurements on PECBs. Last but not the least, the idiosyncratic attributes inherent to various nations, encompassing elements like political structures, societal contexts, and cultural legacies, exert a profound impact on the behaviors exhibited by adolescents. Consequently, a meticulous examination that takes into account the unique characteristics of each nation becomes of paramount importance, thereby highlighting a pivotal trajectory for forthcoming research undertakings. Last but not least, the distinctive attributes inherent to various countries/economies, encompassing elements like political structures, societal contexts, and cultural legacies, profoundly impact adolescents' behaviors. Consequently, a meticulous examination that considers the unique characteristics of each country/economy becomes paramount, thereby highlighting a pivotal trajectory for forthcoming research undertakings.

## 6. Conclusions

In conclusion, our study confirms that all three components of subjective wellbeing, life satisfaction, positive emotions, and negative emotions, are significantly associated with adolescents' PECBs. Moreover, the effect of adolescent subjective wellbeing on PECBs differs substantially between developed and developing countries/economies, and between rural and urban areas. The findings of this paper provide new insight into the challenge of declining pro-environmental behaviors in adolescence.

## Data availability statement

Publicly available datasets were analyzed in this study. This data can be found here: https://www.oecd.org/pisa/data/2018database/.

## Author contributions

MZ: conceptualization, formal analysis, writing—original draft, writing—review and editing, visualization, funding acquisition, and project administration. WZ: formal analysis, data curation, and visualization. YS: conceptualization, formal analysis, writing—original draft, writing—review and editing, funding acquisition, and supervision. All authors contributed to the article and approved the submitted version.
