# Supplementary material for: Can adolescents' subjective wellbeing facilitate their pro-environmental consumption behaviors? Empirical study based on 15-year-old students
Source: Front Public Health. 2023 Oct 5;11:1184605. doi: 10.3389/fpubh.2023.1184605 (PMC10585176; doi:10.3389/fpubh.2023.1184605)
Supplement: Supplementary file 1 [file Table_1.pdf]

## Table 1 Benchmark regression (Bulgaria)

| | PECBs (1) | PECBs (2) | PECBs (3) |
|---|---|---|---|
| ***Life satisfaction*** | -0.038 | | |
| | (-1.12) | | |
| ***Positive emotions*** | | 0.057 | |
| | | (1.63) | |
| ***Negative emotions*** | | | 0.000 |
| | | | (0.01) |
| ***Grade*** | 0.117 | -0.125 | -0.121 |
| | (-1.52) | (-1.61) | (-1.57) |
| ***Gender*** | -0.096* | -0.098* | -0.098* |
| | (-2.37) | (-2.40) | (-2.33) |
| ***Environmental*** | 0.126*** | 0.115*** | 0.122*** |
| ***knowledge*** | (5.87) | (5.41) | (5.78) |
| ***Observations*** | 2,804 | 2,804 | 2,804 |
| ***Pseudo R-squared*** | 0.006 | 0.006 | 0.006 |

*** $p$<0.001, * $p$<0.05, and z-values in parentheses.
