# Supplementary material for: Can adolescents' subjective wellbeing facilitate their pro-environmental consumption behaviors? Empirical study based on 15-year-old students
Source: Front Public Health. 2023 Oct 5;11:1184605. doi: 10.3389/fpubh.2023.1184605 (PMC10585176; doi:10.3389/fpubh.2023.1184605)
Supplement: Supplementary file 2 [file Table_2.pdf]

Table 2 Benchmark regression (Hong Kong)

	PECBs (1)	PECBs (2)	PECBs (3)
Life satisfaction	0.148***		
	(3.83)		
Positive emotions		0.152***	
		(4.72)	
Negative emotions			0.004
			(0.12)
Grade	-0.019	-0.015	-0.020
	(-0.77)	(-0.59)	(-0.82)
Gender	-0.069*	-0.087**	-0.076*
	(-2.23)	(-2.81)	(-2.43)
Environmental knowledge	0.082***	0.076***	0.088***
	(4.02)	(3.74)	(4.33)
Observations	4,852	4,852	4,852
Pseudo R-squared	0.004	0.004	0.002

*** $p<0.001$, ** $p<0.01$, * $p<0.05$, and z-values in parentheses.
