# Supplementary material for: Can adolescents' subjective wellbeing facilitate their pro-environmental consumption behaviors? Empirical study based on 15-year-old students
Source: Front Public Health. 2023 Oct 5;11:1184605. doi: 10.3389/fpubh.2023.1184605 (PMC10585176; doi:10.3389/fpubh.2023.1184605)
Supplement: Supplementary file 3 [file Table_3.pdf]

Table 3 Benchmark regression (Ireland)

	PECBs (1)	PECBs (2)	PECBs (3)
Life satisfaction	0.030		
	(0.76)		
Positive emotions		0.109**	
		(3.25)	
Negative emotions			-0.019
			(-0.57)
Grade	0.002	0.001	0.001
	(0.08)	(0.05)	(0.06)
Gender	0.222***	0.225***	0.227***
	(6.76)	(6.86)	(6.55)
Environmental	0.236***	0.234***	0.237***
knowledge	(12.61)	(12.58)	(12.75)
Observations	4,460	4,460	4,460
Pseudo R-squared	0.019	0.020	0.019

*** p<0.001, ** p<0.01, and z-values in parentheses.
