# Supplementary material for: Can adolescents' subjective wellbeing facilitate their pro-environmental consumption behaviors? Empirical study based on 15-year-old students
Source: Front Public Health. 2023 Oct 5;11:1184605. doi: 10.3389/fpubh.2023.1184605 (PMC10585176; doi:10.3389/fpubh.2023.1184605)
Supplement: Supplementary file 4 [file Table_4.pdf]

Table 4 Benchmark regression (Mexico)

	PECBs (1)	PECBs (2)	PECBs (3)
Life satisfaction	0.164***		
	(4.67)		
Positive emotions		0.221***	
		(6.51)	
Negative emotions			-0.022
			(-0.77)
Grade	-0.175***	-0.168***	-0.169***
	(-3.98)	(-3.80)	(-3.87)
Gender	-0.020	-0.019	-0.029
	(-0.61)	(-0.56)	(-0.84)
Environmental knowledge	0.111***	0.110***	0.123***
	(5.13)	(5.04)	(5.68)
Observations	4,228	4,228	4,228
Pseudo R-squared	0.007	0.009	0.005

*** $p<0.001$, and z-values in parentheses.
