# Supplementary material for: Can adolescents' subjective wellbeing facilitate their pro-environmental consumption behaviors? Empirical study based on 15-year-old students
Source: Front Public Health. 2023 Oct 5;11:1184605. doi: 10.3389/fpubh.2023.1184605 (PMC10585176; doi:10.3389/fpubh.2023.1184605)
Supplement: Supplementary file 5 [file Table_5.pdf]

Table 5 Benchmark regression (Panama)

	PECBs (1)	PECBs (2)	PECBs (3)
Life satisfaction	0.087		
	(1.89)		
Positive emotions		0.163**	
		(3.08)	
Negative emotions			-0.044
			(-0.95)
Grade	-0.183***	-0.181***	-0.177***
	(-3.98)	(-3.95)	(-3.89)
Gender	-0.012	-0.017	-0.006
	(-0.22)	(-0.32)	(-0.11)
Environmental	0.117***	0.115***	0.121***
knowledge	(3.86)	(3.79)	(3.99)
Observations	1,723	1,723	1,723
Pseudo R-squared	0.009	0.010	0.008

*** p<0.001, ** p<0.01, and z-values in parentheses.
