# Supplementary material for: Can adolescents' subjective wellbeing facilitate their pro-environmental consumption behaviors? Empirical study based on 15-year-old students
Source: Front Public Health. 2023 Oct 5;11:1184605. doi: 10.3389/fpubh.2023.1184605 (PMC10585176; doi:10.3389/fpubh.2023.1184605)
Supplement: Supplementary file 6 [file Table_6.pdf]

Table 6 Benchmark regression (Serbia)

	PECBs (1)	PECBs (2)	PECBs (3)
Life satisfaction	0.028		
	(0.85)		
Positive emotions		0.074*	
		(2.18)	
Negative emotions			0.019
			(0.65)
Grade	-0.011	-0.011	-0.011
	(-0.22)	(-0.21)	(-0.21)
Gender	-0.222***	-0.226***	-0.228***
	(-6.30)	(-6.39)	(-6.29)
Environmental	0.084***	0.080***	0.085***
knowledge	(4.51)	(4.33)	(4.57)
Observations	3,742	3,742	3,742
Pseudo R-squared	0.007	0.007	0.006

*** p<0.001, * p<0.05, and z-values in parentheses.
