# Supplementary material for: Can adolescents' subjective wellbeing facilitate their pro-environmental consumption behaviors? Empirical study based on 15-year-old students
Source: Front Public Health. 2023 Oct 5;11:1184605. doi: 10.3389/fpubh.2023.1184605 (PMC10585176; doi:10.3389/fpubh.2023.1184605)
Supplement: Supplementary file 7 [file Table_7.pdf]

## Table 7 Benchmark regression (Spain)

| | PECBs (1) | PECBs (2) | PECBs (3) |
|---|---|---|---|
| ***Life satisfaction*** | 0.056** | | |
| | (3.48) | | |
| ***Positive emotions*** | | -0.004 | |
| | | (-0.25) | |
| ***Negative emotions*** | | | 0.035* |
| | | | (2.21) |
| ***Grade*** | -0.033* | -0.030 | -0.032* |
| | (-2.16) | (-1.95) | (-2.06) |
| ***Gender*** | -0.025 | -0.030* | -0.039* |
| | (-1.66) | (-1.99) | (-2.52) |
| ***Environmental*** | 0.161*** | 0.163*** | 0.163*** |
| ***knowledge*** | (19.33) | (19.65) | (19.68) |
| ***Observations*** | 20,977 | 20,977 | 20,977 |
| ***Pseudo R-squared*** | 0.008 | 0.008 | 0.008 |

*** $p$<0.001, ** $p$<0.01, * $p$<0.05, and z-values in parentheses.
