# Supplementary material for: Can adolescents' subjective wellbeing facilitate their pro-environmental consumption behaviors? Empirical study based on 15-year-old students
Source: Front Public Health. 2023 Oct 5;11:1184605. doi: 10.3389/fpubh.2023.1184605 (PMC10585176; doi:10.3389/fpubh.2023.1184605)
Supplement: Supplementary file 8 [file Table_8.pdf]

Table 8 Benchmark regression (United Arab Emirates)

	PECBs (1)	PECBs (2)	PECBs (3)
Life satisfaction	0.272***		
	(16.05)		
Positive emotions		0.211***	
		(12.93)	
Negative emotions			-0.154***
			(-11.13)
Grade	-0.025*	-0.029*	-0.021
	(-1.99)	(-2.30)	(-1.69)
Gender	-0.105***	-0.109***	-0.065***
	(-5.72)	(-5.97)	(-3.49)
Environmental	0.016	0.016	0.037***
knowledge	(1.67)	(1.68)	(3.85)
Observations	14,396	14,396	14,396
Pseudo R-squared	0.009	0.006	0.005

*** p<0.001, * p<0.05, and z-values in parentheses.
